# Seeing voices? The importance of raising awareness on Deaf Mental Health

**DOI:** 10.1192/j.eurpsy.2023.1914

**Published:** 2023-07-19

**Authors:** M. Conde Moreno, F. Ramalheira, M. Couto Bártolo

**Affiliations:** 1 Centro hospitalar Psiquiátrico de Lisboa, Lisboa; 2 Interna formação Geral, Torres Vedras, Portugal

## Abstract

**Introduction:**

Data regarding mental health problems in the prelingual deaf population is scarce. There is evidence that factors related to minority stress can contribute to mental illness in that population. In psychiatry, communication is key, however, most clinicians are not trained to communicate with the Deaf. Moreover, psychiatrists are often not aware of particularities in the psychopathology of these patients.

**Objectives:**

We aim to review important aspects of psychiatric evaluation of prelingual Deaf patients.

**Methods:**

Non-systematic review of recent literature regarding Deaf mental health and mental illness.

**Results:**

Data regarding prevalence of mental illness in the Deaf population is mostly obtained from small studies and suggest an increased burden of mental illness and significant barriers to mental health care. Psychiatry research regarding Deaf patients is about 40 years behind research on the hearing population. While communicating with a Deaf patient, clinicians should consider the preferred communication modality. Sign-language interpreters should have specific mental health training, although that is not the case for many countries. Clinicians should keep communication simple, use short sentences, concrete examples and visual aids.

The mental status examination will have particularities, such as: 1) facial expressions have a specific role in sign languages and may not relate to affect; 2) There is a need to distinguish between language dysfluency and thought disorder 3) voice hallucinations may manifest as somatic or visual hallucinations; the occurrence of pure auditory hallucination in the prelingual Deaf is controversial. 4) the Deaf have little access to health information and are likely to demonstrate poor literacy on mental health matters.

**Conclusions:**

More studies regarding the mental health issues of the Deaf population should be conducted. Raising awareness among clinicians about the needs of Deaf population is an important step to improve their access to help and treatment.

**Disclosure of Interest:**

None Declared

